# Two-Week Isocaloric Time-Restricted Feeding Decreases Liver Inflammation without Significant Weight Loss in Obese Mice with Non-Alcoholic Fatty Liver Disease

**DOI:** 10.3390/ijms21239156

**Published:** 2020-12-01

**Authors:** Rachel B. Wilson, Richard Zhang, Yun Jin Chen, Kia M. Peters, Cynthia G. Sawyez, Brian G. Sutherland, Krisha Patel, John P. Kennelly, Kelly-Ann Leonard, René L. Jacobs, Rennian Wang, Nica M. Borradaile

**Affiliations:** 1 Department of Physiology and Pharmacology, Schulich School of Medicine and Dentistry, Western University, London, ON N6A 5C1, Canada; rwilso89@uwo.ca (R.B.W.); rzhang2023@meds.uwo.ca (R.Z.); yche922@uwo.ca (Y.J.C.); kpeter44@uwo.ca (K.M.P.); csawyez@uwo.ca (C.G.S.); bsuther2@uwo.ca (B.G.S.); kpate253@uwo.ca (K.P.); rwang@uwo.ca (R.W.); 2 Group on the Molecular and Cell Biology of Lipids, Department of Agricultural, Food and Nutritional Science, University of Alberta, Edmonton, AB T6G 2R3, Canada; jkennell@ualberta.ca (J.P.K.); kmd4@ualberta.ca (K.-A.L.); rjacobs@ualberta.ca (R.L.J.); 3 Department of Pathology and Laboratory Medicine, Schulich School of Medicine and Dentistry, Western University, London, ON N6A 5C1, Canada

**Keywords:** NAFLD, liver, inflammation, obesity, mouse

## Abstract

Prolonged, isocaloric, time-restricted feeding (TRF) protocols can promote weight loss, improve metabolic dysregulation, and mitigate non-alcoholic fatty liver disease (NAFLD). In addition, 3-day, severe caloric restriction can improve liver metabolism and glucose homeostasis prior to significant weight loss. Thus, we hypothesized that short-term, isocaloric TRF would improve NAFLD and characteristics of metabolic syndrome in diet-induced obese male mice. After 26 weeks of ad libitum access to western diet, mice either continued feeding ad libitum or were provided with access to the same quantity of western diet for 8 h daily, over the course of two weeks. Remarkably, this short-term TRF protocol modestly decreased liver tissue inflammation in the absence of changes in body weight or epidydimal fat mass. There were no changes in hepatic lipid accumulation or other characteristics of NAFLD. We observed no changes in liver lipid metabolism-related gene expression, despite increased plasma free fatty acids and decreased plasma triglycerides in the TRF group. However, liver *Grp78* and *Txnip* expression were decreased with TRF suggesting hepatic endoplasmic reticulum (ER) stress and activation of inflammatory pathways may have been diminished. We conclude that two-week, isocaloric TRF can potentially decrease liver inflammation, without significant weight loss or reductions in hepatic steatosis, in obese mice with NAFLD.

## 1. Introduction

Treatment strategies for non-alcoholic fatty liver disease (NAFLD) encompass behavioural, pharmacological, and surgical approaches primarily focused on improving blood glucose, triglycerides, and cholesterol—the metabolic parameters associated with this disease [1,2]. Behavioural modifications aimed at weight loss are effective in improving many components of metabolic syndrome [3], however thresholds of 5–10% and >10% weight loss are needed to reduce steatosis and improve non-alcoholic steatohepatitis (NASH), respectively [3]. To this end, bariatric surgery has become an increasingly common treatment for obesity and its complications. Gastric bypass, in particular, can be effective in resolving NASH and fibrosis, but the safety and long-term efficacy of this approach are not yet firmly established [3]. In the absence of bariatric surgery, achieving sufficient weight loss can be challenging for individuals with obesity-associated conditions that may limit physical activity. In such cases, alternative dietary protocols could be especially useful. Time-restricted feeding (TRF) protocols, including intermittent fasting, in which food consumption is restricted to specific times in the day, have garnered intense public interest as potential treatments for obesity and obesity-related diseases [4].

To date, evidence of the clinical benefit of time-restricted feeding on NAFLD is scarce. However, alternate-day caloric restriction (a form of time-restricted feeding) has been shown to improve liver steatosis and fibrosis in subjects with metabolic syndrome and NAFLD [5], and an ongoing trial of Time Restricted Feeding on Nonalcoholic Fatty Liver Disease (TREATY-FLD) has been registered with CinicalTrials.gov. In mice, prolonged, isocaloric TRF can prevent obesity, metabolic dysregulation, and hepatic steatosis [6,7,8]. Recent work has further shown that 3-day, severe caloric restriction improves liver metabolism and glucose homeostasis prior to weight loss [9]. However, studies of the effects of TRF intervention on obesity and NAFLD severity in rodents have been less conclusive [8,10]. Moreover, the most commonly used mouse strains for these types of studies, C57BL/6, do not develop consistent, significant liver fibrosis [11,12], which is the strongest predictor of mortality in NAFLD [3,13]. Considering this previous work, the effects of TRF, and of short-term isocaloric TRF in particular, on NAFLD progression remain to be determined. The 129S6 mouse strain has been shown to develop fibrosis with prolonged high fat feeding and so is an attractive model for investigating obesity-associated NAFLD with potential progression to fibrosis [14].

We therefore hypothesized that short-term, isocaloric TRF would diminish NAFLD severity and improve metabolic parameters associated with liver function. To test this hypothesis, male 129S6 mice were provided ad libitum access to western diet for 26 weeks. Following diet induction of obesity, mice either continued feeding ad libitum or were provided with access to the same quantity of western diet for 8 h daily, at the beginning of the inactive phase (8:00 AM), over the course of two weeks. This pattern of TRF is similar to dawn-to-sunset Ramadan fasting in humans. We subsequently measured metabolic parameters associated with metabolic syndrome and NAFLD, histopathological characteristics of NAFLD, and hepatic expression of endoplasmic reticulum (ER) stress and inflammatory genes which are associated with the development and progression of this disease [15].

## 2. Results

### 2.1. Two-Week TRF Does Not Promote Weight Loss or Resolution of Hepatic Steatosis

**Figure 1:**
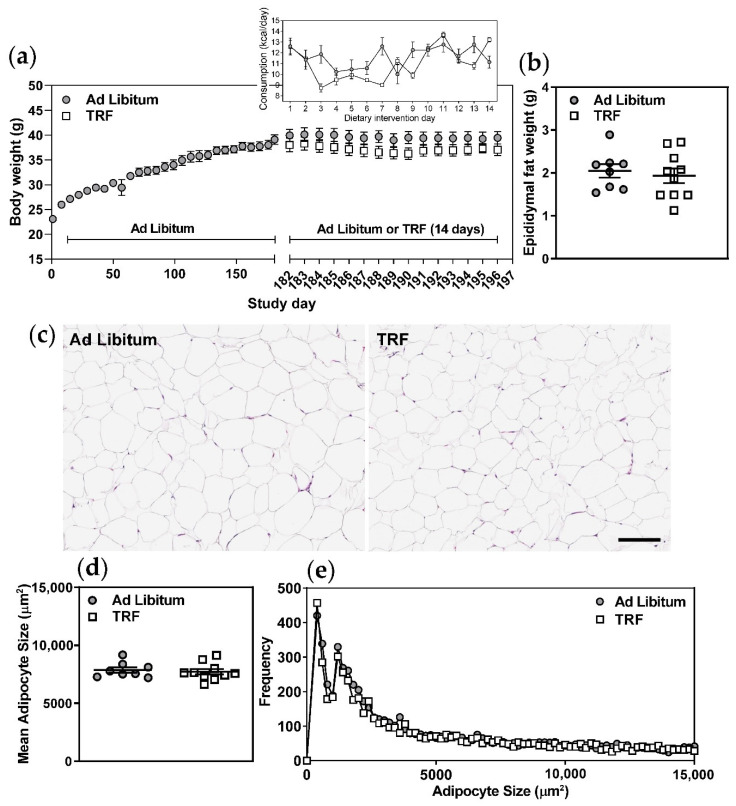
Two-week TRF does not significantly alter body weight, adipocyte size, or adipocyte size distribution in obese mice. (**a**) Body weights and food consumption (inset) were recorded on the indicated days. (**b**) Epididymal fat pads were excised and weighed post mortem. (**c**) Epidydimal adipose tissue sections were stained with hematoxylin and eosin (HE) to visualize tissue morphology. (**d**,**e**) Images were analyzed using AdipoCount to determine adipocyte number per image and individual adipocyte areas (adipocyte size). Mean adipocyte areas were calculated and adipocyte size frequencies were plotted. Scale bar represents 100 µm, *n* = 8–10.

**Figure 2:**
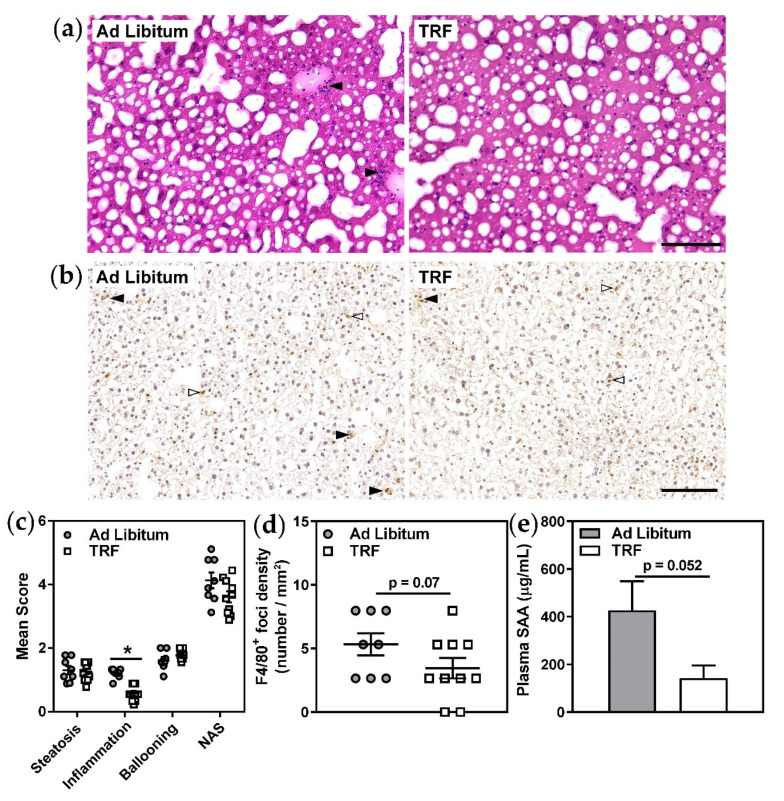
Two-week TRF appears to decrease liver inflammation in obese mice. (**a**) Liver tissue sections were stained with HE to visualize tissue morphology and inflammatory foci (black arrows). (**b**) Macrophages were visualized by immunohistochemistry for the pan-macrophage marker F4/80. Inflammatory foci, identified as clusters of F4/80-positive cells with small, rounded nuclei (black arrows), were counted. Kupffer cells, identified as F4/80-positive cells with single large nuclei (white arrows), were excluded from counting. (**c**) Steatosis, lobular inflammation, ballooning, and NAFLD activity scores (NAS) were assessed in HE-stained serial sections. (**d**) F4/80-positive inflammatory foci densities were determined per section area. (**e**) Plasma serum amyloid A (SAA) was measured by ELISA. Scale bars represent 100 µm. Data are means ± SEM, *n* = 8–10, * *p* < 0.05.

Isocaloric TRF for 14 days did not result in significant changes in body weight, liver wet weight, liver lipids, plasma ALT, AST, or albumin (Table 1 and Figure 1a). Although food consumption was variable in both experimental groups from day to day (Figure 1a, inset), mean daily caloric intake between groups was not significantly different over the 14-day course of the feeding intervention protocol (Table 1). Daily body weights (Figure 1a) and wet epididymal fat weights measured post-mortem (Figure 1b) were not significantly different between experimental groups. Histological assessments of epidydimal adipose tissue sections showed no changes in mean adipocyte size or size frequency in the TRF group versus ad libitum-fed control mice (Figure 1c–e). Histological analyses of hematoxylin and eosin (HE)-stained liver sections (Figure 2a,c) were consistent with biochemical analyses of liver lipid content (Table 1), further indicating no change in hepatic steatosis in the TRF group. The lack of inclusion of lean control groups is a limitation of our study design, however, body composition, metabolic and liver parameters for lean, chow-fed 129S6 mice of the same age as those used in the current work can be found in our recent publication [16].

### 2.2. Two-Week TRF Appears to Improve Hepatic Inflammation

**Figure 3:**
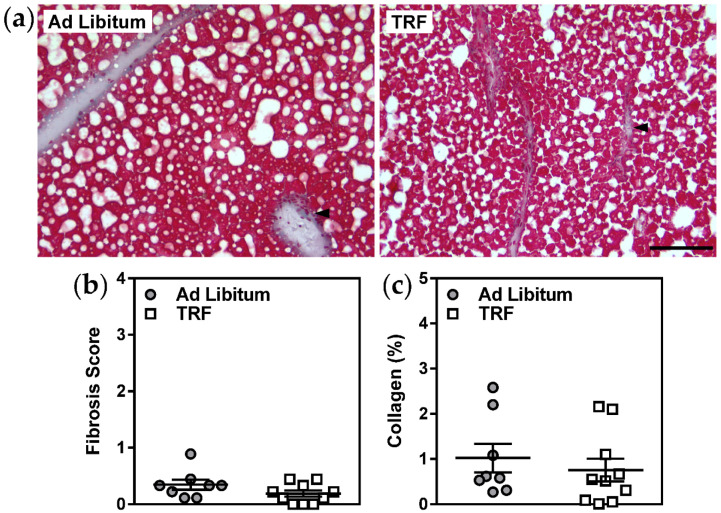
Two-week TRF does not alter liver fibrosis in obese mice. (**a**) Liver tissue sections were stained with Masson’s trichrome to visualize tissue fibrosis (collagen; blue, black arrows). (**b**,**c**) Fibrosis scores (from 0 to 4) and collagen tissue areas were assessed in 3 serial sections per mouse. Scale bar represents 100 µm. Data are means ± SEM, *n* = 8–10.

Remarkably, analyses of HE-stained sections revealed a specific and significant decrease in the number of inflammatory foci in the livers of TRF mice (Figure 2a,c). Immunostaining of inflammatory foci for the pan-macrophage marker F4/80, although more challenging to quantitate due to the abundance of Kupffer cells in fatty livers, also appeared to be generally consistent with this observation (Figure 2b,d). This apparent decrease in lobular inflammation in TRF mice was accompanied by decreased concentrations of plasma SAA (Figure 2e), an acute-phase protein which originates from liver, further supporting the concept that short-term isocaloric TRF improves hepatic inflammation in obese mice with NAFLD. Moreover, liver gene expression of the ER-resident protein folding chaperone, *Grp78*, and of the inflammasome activating factor, *Txnip*, were decreased with TRF suggesting that hepatic ER stress and subsequent activation of inflammatory pathways may be diminished with this feeding protocol (Table 2). Other ER stress-responsive genes and inflammatory genes were not altered. Additional characteristics of NAFLD, including hepatocellular ballooning (Figure 2c), fibrosis score, and collagen content (Figure 3), were unaffected by two-week isocaloric TRF.

### 2.3. Two-Week TRF Decreases Blood Glucose but Does Not Improve Systemic Glucose Homeostasis

**Figure 4:**
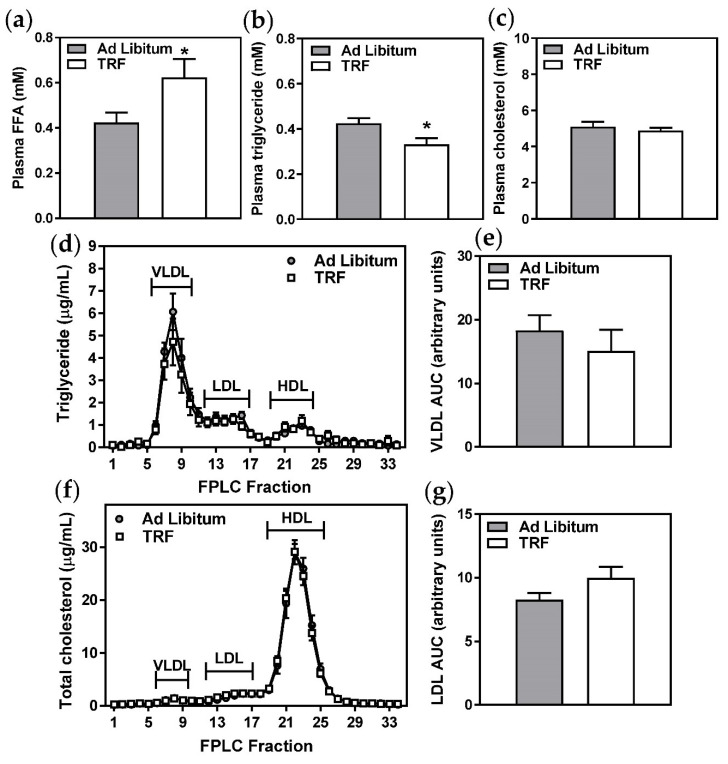
Two-week TRF increases circulating FFA without altering lipoprotein profiles. (**a**–**c**) Total plasma free fatty acids (FFA), triglyceride, and cholesterol were measured using standard enzymatic, colorimetric assays. Data are means ± SEM, *n* = 8–10, * *p* < 0.05. (**d**–**g**) Triglyceride and cholesterol concentrations were measured in very low-density lipoprotein (VLDL), low density lipoprotein (LDL), and high-density lipoprotein (HDL) eluted plasma fractions. Areas under the curve (AUC) for VLDL triglyceride and LDL cholesterol were calculated. Data are means ± SEM, *n* = 6.

**Figure 5:**
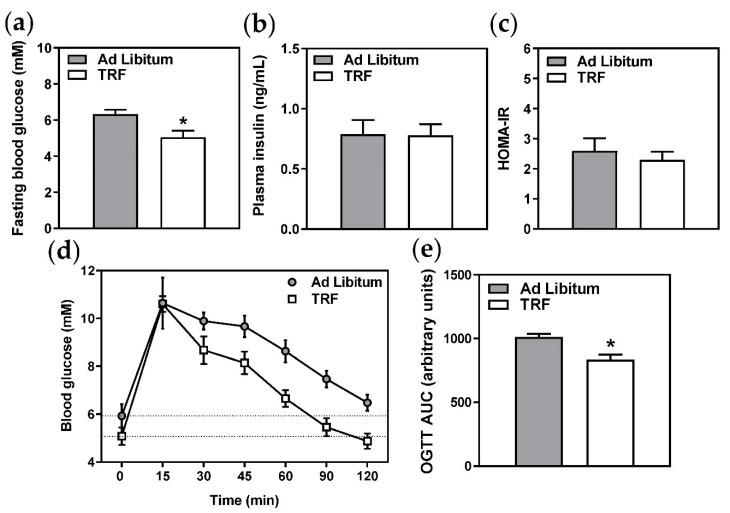
Two-week TRF decreases fasting blood glucose and improves oral glucose tolerance, but does not affect systemic glucose homeostasis. (**a**) Fasting blood glucose was measured immediately prior to sacrifice by handheld glucometer. (**b**) Plasma insulin was measured by ELISA. (**c**) HOMA-IR was calculated using blood glucose and plasma insulin values. (**d**) Glucose tolerance after oral gavage of 1 g glucose/kg body weight was measured following a 6 h fast for the ad libitum group and an approximately 22 h fast for the TRF group on the day prior to sacrifice (day 14). (**e**) Areas under the curve were calculated from data in (**d**). Data are means ± SEM, *n* = 8–10, * *p* < 0.05.

**Figure 6:**
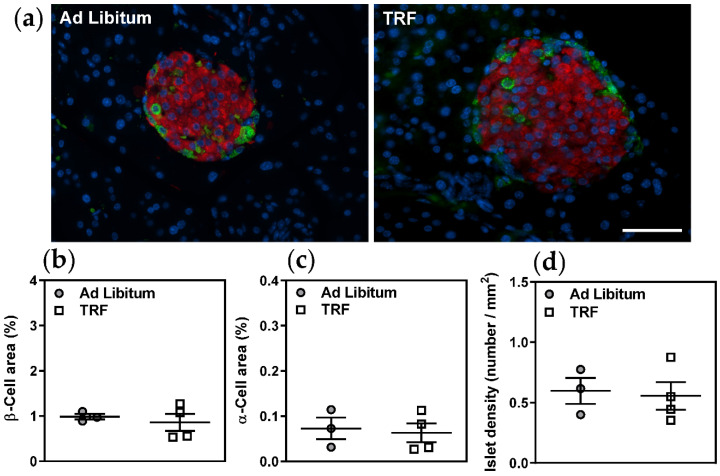
Two-week TRF does not alter pancreatic islet architecture in obese mice. (**a**) β-cells and α-cells were visualized by immunofluorescent staining for insulin (red) and glucagon (green), respectively. Nuclei were counterstained with DAPI (blue). (**b**,**c**) β-cell and α-cell areas were determined per total pancreas areas. (**d**) Islet densities were determined per total tissue area. Scale bar represents 50 µm. Data are means ± SEM, *n* = 3–4.

As expected due to the longer duration of fast, plasma free fatty acids were increased in the TRF group (Figure 4a) and plasma triglycerides were decreased (Figure 4b). Plasma cholesterol and lipoprotein profiles were not significantly altered (Figure 4c–g), and liver lipid metabolism-related gene expression was similarly unaffected (Table 2). However, fasting blood glucose (Figure 5a) and oral glucose tolerance, indicated by significantly decreased area under the curve (Figure 5c,d), were improved with TRF. These observations were not unexpected given the extended daily fasting time of the TRF group, which resulted in a longer fast immediately prior to oral glucose tolerance tests and sacrifice. However, given that the fasting plasma insulin (Figure 5b), HOMA-IR (Figure 5c), and pancreatic islet morphology (Figure 6a–c) and density (Figure 6d) were unchanged, two-week TRF appeared not to significantly improve systemic glucose homeostasis.

## 3. Discussion

Liver steatosis is the first step in the pathogenesis of NAFLD/NASH, as it can lead to sustained hepatic inflammation through hepatocyte insulin resistance, ER stress, and progressive cellular dysfunction [3,15]. Thus, we were intrigued by our observations which appear to be consistent with modestly decreased lobular inflammation in response to two-week TRF, while hepatic steatosis and plasma lipoprotein profiles were unchanged. Further observations of decreased liver *Grp78* and *Txnip* expression suggested that hepatic ER stress was diminished with TRF. Although other ER stress-responsive genes (*Eif2ak3*, *Atf6*, *Chop*, and *Xbp1s*) were not altered with TRF, these factors should be assessed at the protein level and are a direction for future investigation. Since *Txnip* is a known effector of hepatocyte inflammasome activation in response to ER stress [15], this is a possible mechanism for TRF-mediated reductions in lobular inflammation that also warrants further investigation of inflammatory mediators at the protein level. Overall, our current observations supported our hypothesis that short-term, isocaloric TRF would diminish NAFLD severity and improve metabolic parameters associated with liver function.

Long-term intermittent fasting and gastric bypass surgery have been shown to decrease hepatic ER stress [17] and inflammation [7], markers of disease progression [15], in association with decreased liver steatosis and body weight reduction. However, our data suggest that some of these benefits may occur early, and prior to significant reductions in body weight or hepatocyte lipid accumulation. Furthermore, these benefits were evident with two-week TRF occurring at the beginning of the inactive phase for mice (beginning of the light cycle). These findings are distinct from observations of metabolic dysfunction with longer-term inactive phase TRF [18], but are consistent with benefits which have been reported in dawn-to-sunset Ramadan fasting studies [19], in which the feeding pattern is similar to the TRF protocol used for our study. Thus our observations suggest that the length of the fast alone may be sufficient to ameliorate hepatic inflammation.

Another possible explanation for decreased lobular inflammation with short-term TRF relates to our observations of improved oral glucose tolerance. This effect on glucose tolerance was likely acute and/or cyclic in nature in our model as a result of periodic fasting. Since we observed no changes in fasting insulin concentrations and HOMA-IR, it is unlikely that systemic glucose homeostasis was improved in any sustained way, which is consistent with previous studies of TRF occurring in the inactive phase [18]. Previous studies have shown that restoring peripheral insulin sensitivity and glucose homeostasis can reverse the pathogenesis of NAFLD by normalizing adipokine and cytokine secretion from adipose tissue [20]. However, the contribution of adipose-derived cytokine production to glucose intolerance in obese mice has recently been challenged [21]. Our studies cannot exclude the possibility that TRF decreased lobular inflammation by improving insulin sensitivity at adipose tissue, and subsequently reducing adipocyte production of inflammatory cytokines. However, our analyses of epididymal adipose suggest that two-week TRF did not significantly affect this tissue. We speculate that TRF altered the frequency and amount of fatty acid delivery to the liver, thereby locally and periodically improving hepatic insulin sensitivity and glucose regulation [6,9]. This concept is consistent with the less direct supply of free fatty acids originating primarily from adipose lipolysis in the TRF mice, as compared to the unrestricted dietary supply in the ad libitum-fed mice.

Fibrosis was not significantly affected by short-term TRF in our study. This observation was partly due to the modest fibrosis we observed in our model, which was unexpected based on previous work [14], and limited our ability to detect changes. However, it is also likely that a more prolonged TRF protocol and concomitant prolonged suppression of hepatic inflammation would be required to prevent progression and/or resolve fibrosis, as has been demonstrated for specific anti-inflammatory interventions [2,22]. Similarly, the duration of TRF was likely too short for potential improvements in parameters other than lobular inflammation, such as hepatocyte ballooning, to be detected. Future investigations using a model of advanced NAFLD (e.g., extended duration of western diet feeding and/or supplementation with fructose) with comparisons of disease development in age-matched lean control mice and different TRF protocols are warranted.

Considering current public interest in TRF for the potential treatment of obesity and its complications [4], it is important to understand the short- and long-term metabolic and physiological consequences of these alternative dietary patterns. If translatable to humans, our finding that two weeks of isocaloric TRF can potentially improve hepatic ER stress and inflammation, prior to significant weight loss or reductions in hepatic steatosis, could be clinically useful. Individuals with progressive NAFLD, who struggle to maintain long-term optimal eating behaviours, could still potentially benefit from short-term TRF. Given the recently elucidated links between hepatic ER stress, NASH, and the development of hepatocellular carcinoma [23], this is an encouraging possibility for an approachable behavioural modification to complement emerging pharmacological therapies.

## 4. Materials and Methods

### 4.1. Obese Mouse Model and Time-Restricted Feeding Protocol

All mouse experiments were approved by the Animal Use Subcommittee at Western University (protocol 2017-158) in accordance with Canadian Council on Animal Care guidelines. Twenty 5-week-old male 129S6 mice purchased from Taconic (Germantown, NY, USA) were fed Western diet (TD.88137, Envigo, Toronto, ON, Canada) containing 42% kcal from fat ad libitum for 26 weeks. This protocol is known to cause obesity and NAFLD with fibrosis [14]. During the final 7 weeks of ad libitum feeding, mice were housed individually. For the following 2 weeks, 10 mice were randomly assigned to time-restricted feeding of Western diet by receiving the average daily allotment of diet at 8:00 AM each morning. Thus, food was provided at the beginning of the light cycle, which was the beginning of the inactive phase, with any remaining food being removed at 4:00 PM. This pattern of TRF is similar to dawn-to-sunset Ramadan fasting in humans, during which food is consumed at the end of the active time of day. The remaining 10 mice continued to feed ad libitum. Mice assigned to the TRF protocol gorged when the meal was provided, finishing it in the mornings (beginning of the inactive phase), when they learned that they would not receive additional food. Measurements of food remaining in the cages of ad libitum-fed mice confirmed that this protocol resulted in no significant differences in caloric intake between the two groups. Two mice in the ad libitum-fed group failed to gain significant weight over the 196-day course of the study and were excluded from all subsequent analyses. This study was conducted concurrently with another pharmacological intervention study [16], such that the group which remained on ad libitum feeding of Western diet was shared between these two studies. At the conclusion of the study, all mice were euthanized by carbon dioxide inhalation.

### 4.2. Glucose Homeostasis, Euthanasia, and Tissue Collection

On day 14 of the 2-week feeding protocol intervention, all mice were fasted for 6 h, beginning at 8:00 AM, and a 2 h glucose tolerance test was performed with an Ascensia Elite glucometer (Bayer, Mississauga, ON, Canada) following oral glucose gavage (1 g of 20% glucose/kg body weight). The following day, mice were again fasted for 4 h prior to measurements of blood glucose, followed by carbon dioxide euthanasia and immediate collection of blood, adipose, pancreas, and liver tissues. Plasma insulin was subsequently measured using a mouse ultrasensitive insulin ELISA (Alpco, Salem, NH, USA).

### 4.3. Liver Lipids and Enzymes

Total liver triglycerides and cholesterol were extracted from liver samples using the Folch method, solubilized, and measured by enzymatic, spectrophotometric assays (Wako Diagnostics, Richmond, VA, USA) [24]. Plasma alanine aminotransferase (ALT) and aspartate aminotransferase (AST) were determined by enzymatic rate assays (Roche Diagnostics, Laval, QC, Canada) and plasma albumin was determined by spectrophotometric assay (Roche Diagnostics) at the Animal Health Laboratory at the University of Guelph. Serum amyloid A (SAA) was measured in plasma by ELISA (Tridelta Development Limited, Maynooth, Ireland).

### 4.4. Histology

#### 4.4.1. Adipose

Epididymal adipose tissue samples were fixed with 4% paraformaldehyde, embedded in paraffin, sectioned at 5 µm, and stained with hematoxylin and eosin (Leica Biosystems, Buffalo Grove, IL, USA). Brightfield images of sections were captured at 20X magnification using an Aperio AT2 whole slide scanner (Leica Biosystems) within the Molecular Pathology Core at Robarts Research Institute, Western University. Images were analyzed using AdipoCount [25] which determines adipocyte number and size (area) by segmenting clear (white) areas of the image from stained cell membrane edges. Manual corrections were performed following automated segmentation to remove areas containing tissue processing artifacts, and to add segmentations to areas of weak membrane staining which were not captured through automated segmentation. All image analyses were randomized and performed blind to treatment group.

#### 4.4.2. Liver

Liver samples were embedded in optimal cutting temperature compound (Sakura Finetek, Maumee, OH, USA) and stored at −80 °C. Hepatic sections (8 µm) were prepared by cryostat (Leica Biosystems). Three sections per mouse were stained with hematoxylin and eosin (Leica Biosystems) to assess steatosis, lobular inflammation, and hepatocellular ballooning, and with Masson’s trichrome (Leica Biosystems) to assess fibrosis. Three brightfield images per section were captured at magnifications of 10× (for evaluation of steatosis) and 20× (for evaluation of lobular inflammation and hepatocyte ballooning) using an Olympus BX51 microscope (Molecular Pathology Core, Robarts Research Institute, Western University, London, ON, Canada) for a total of nine fields of view per stain per mouse [26].

The NASH-CRN scoring method [27] was used to assess steatosis, lobular inflammation, and hepatocellular ballooning, as we have done previously [26]. Image analyses were randomized and performed blind to treatment group. Hematoxylin and eosin images were used to score steatosis, lobular inflammation, and hepatocellular ballooning. The percent of the image that consisted of lipid droplets (% steatosis) was quantified using an ImageJ macro developed in house. This macro determines lipid droplet area by segmenting clear (white) areas of the section from stained areas using an intensity threshold. Clear areas not corresponding to lipid droplets, such as vasculature, were manually excluded from the measurement. Steatosis was scored from 0–3 using the calculated % steatosis (0: <5%; 1: 5–33%; 2: 34–66%; 3: ≥67%). Ballooned hepatocytes were defined as enlarged, rounded hepatocytes with pale, rarified cytoplasm. Hepatocellular ballooning was scored from 0–2 based on the frequency and severity of ballooned hepatocytes (0: none; 1: few ballooned hepatocytes; 2: many ballooned hepatocytes and/or prominent ballooning). Lobular inflammation was scored from 0–3 based on the number of inflammatory foci, defined as clusters of greater than 5 small, rounded nuclei (0: none; 1: one focus; 2: 2–4 foci; 3: >4 foci). Scores for steatosis, hepatocellular ballooning, and lobular inflammation were summed to generate an aggregate NAFLD activity score (NAS).

Masson’s trichrome images were used to evaluate liver fibrosis by two approaches. Fibrosis was scored from 0–4 based on fibrosis location using the NASH-CRN method (0: no fibrosis; 1: periportal or perisinusoidal fibrosis; 2: both periportal and perisinusoidal fibrosis; 3: bridging fibrosis; 4: cirrhosis). Additionally, the percent of the image that was collagen-positive (% collagen) was quantified using an ImageJ macro developed in house [28]. This macro segments and measures the collagen-positive area (blue) by performing a color deconvolution to separate the colors in the Masson’s trichrome stain, followed by application of an intensity threshold.

Liver inflammation was further confirmed by immunostaining for the mouse pan-macrophage marker, F4/80. Hepatic sections were incubated with rat monoclonal anti-F4/80 (dilution 1:50, Invitrogen), and subsequently counterstained with hematoxylin (Leica Biosystems). Brightfield images of sections were captured at 20X magnification using an Aperio AT2 whole slide scanner (Leica Biosystems). To distinguish between resident Kupffer cells and inflammatory foci [29], clusters of greater than 5 F4/80-positive cells with small nuclei were counted in one field of view per section per mouse. Kupffer cells, identified as F4/80-positive cells with single large nuclei [29], were excluded from counting as it is not necessarily the number but the balance between pro- and anti-inflammatory phenotype of these cells which determines their contribution to lobular inflammation [30]. Inflammatory foci densities were expressed per section area.

#### 4.4.3. Pancreas

Pancreatic tissue samples were fixed with 4% paraformaldehyde, embedded in paraffin, and sectioned at 4 µm for subsequent immunofluorescence microscopy. Sections were incubated with the primary antibodies: rabbit polyclonal anti-glucagon (dilution 1:100, Sigma-Aldrich, St. Louis, MO, USA), mouse monoclonal anti-insulin (dilution 1:800, Sigma-Aldrich) or rabbit polyclonal anti-insulin (dilution 1:100, Cell Signaling Technology, Beverly, MA, USA), followed by FITC-conjugated and TRITC-conjugated secondary antibodies (Jackson Immunoresearch, West Grove, PA, USA). Nuclei were counterstained with 4′,6-diamidino-2-phenylindole (DAPI). Images of at least 8 islets per pancreas were captured at 40X magnification using a DMIRE2 OPTI-tech fluorescence microscope (Leica Biosystems). Insulin-positive and glucagon-positive areas were outlined using Image ProPlus software to measure β-cell areas and α-cell areas, respectively. Total pancreatic section areas were determined from images obtained by bright field microscopy at 10X magnification. The number of islets per total pancreatic area (mm^2^) (islet density), and β-cell and α-cell areas normalized to total pancreas areas were calculated as previously described [31].

### 4.5. Plasma Lipids and Lipoproteins

Total plasma triglyceride, total cholesterol, and free fatty acid concentrations were determined by spectrophotometric assays (Wako Diagnostics, Richmond, VA, USA). Plasma lipoprotein distribution was determined by fast performance liquid chromatography (FPLC) [24]. Fresh EDTA plasma was separated by FPLC using an AKTA purifier (GE Healthcare Life Sciences, Mississauga, ON, Canada) and a Superose 6 column (GE Healthcare Life Sciences). Total cholesterol and triglyceride contents of collected fractions were determined using spectrophotometric assays (Wako Diagnostics and Roche Diagnostics, respectively).

### 4.6. Gene Expression

Expression of a panel of genes relevant to metabolism, cellular stress, and inflammation was analyzed as described previously [16,32]. Total RNA was isolated from liver using Trizol (Invitrogen, Carlsbad, CA, USA). RNA was reverse-transcribed using Superscript II (Invitrogen). Quantitative PCR was run on an Applied Biosystems StepOne Plus for 40 cycles using a Power SYBR Green PCR Master Mix (Applied Biosystems, Foster City, CA, USA), in triplicate. Relative mRNA expression was normalized to *Rplp0* and expression for each gene of interest was calculated using the standard curves method.

### 4.7. Statistical Analyses

All area under the curve calculations and statistical analyses were performed using GraphPad Prism 6.0c (GraphPad Software, San Diego, CA, USA). All metabolic parameters, histological features, and pathophysiological scores were analyzed by unpaired, two-tailed Student’s t-test.

## Figures and Tables

**Table 1:** Biochemical parameters of non-alcoholic fatty liver disease (NAFLD) in experimental mouse groups ^1^.

Feeding Protocol	Ad Libitum	TRF
Number of mice	8	10
Mean daily food intake (kcal/day)	11.6 ± 0.4	10.9 ± 0.1
Liver weight (g)	1.36 ± 0.06	1.25 ± 0.03
Liver triglycerides (mg/g)	178.6 ± 11.7	177.0 ± 10.6
Liver cholesterol esters (mg/g)	11.06 ± 0.93	11.28 ± 0.95
Liver free cholesterol (mg/g)	2.90 ± 0.44	2.56 ± 0.28
Plasma ALT (U/L)	24.4 ± 2.1	28.4 ± 8.6
Plasma AST (U/L)	67.4 ± 2.7	93.6 ± 20.0
Plasma albumin (g/L)	32.5 ± 0.6	34.0 ± 0.5

^1^ Diet-induced obese male 129S6 mice were fed western diet either ad libitum or on a time-restricted feeding (TRF) protocol for 14 days. Food intake was measured daily during the feeding protocol intervention period, and mean food intake per day over the course of the intervention was calculated. All other parameters were measured post-mortem.

**Table 2:** Liver tissue gene expression ^1^.

Feeding Protocol	Ad Libitum	TRF
Lipid and lipoprotein metabolism		
*Acox1*	0.84 ± 0.06	0.77 ± 0.06
*Cpt1a*	0.77 ± 0.06	0.79 ± 0.08
*Ucp2*	0.79 ± 0.06	0.69 ± 0.06
*Cyp7a1*	0.90 ± 0.13	1.00 ± 0.11
*Bsep*	0.75 ± 0.08	0.61 ± 0.04
*Fgf21*	0.33 ± 0.08	0.23 ± 0.04
*Hmgcr*	0.76 ± 0.08	0.81 ± 0.06
*Ldlr*	0.90 ± 0.08	0.93 ± 0.08
*Apoa1*	0.73 ± 0.04	0.71 ± 0.04
ER homeostasis		
*Grp78*	1.20 ± 0.13	0.79 ± 0.12 *
*Eif2ak3*	1.14 ± 0.08	1.04 ± 0.06
*Atf6*	0.76 ± 0.06	0.68 ± 0.06
*Chop*	0.67 ± 0.04	0.59 ± 0.04
*Xbp1s*	0.50 ± 0.06	0.48 ± 0.12
*Txnip*	0.94 ± 0.13	0.84 ± 0.04 *
Inflammation and fibrosis		
*Cd68*	1.09 ± 0.07	0.93 ± 0.11
*Emr1*	1.26 ± 0.07	1.22 ± 0.16
*Mcp1*	0.92 ± 0.17	0.62 ± 0.14 ^†^
*Tnfa*	0.91 ± 0.12	0.86 ± 0.16
*Fn1*	0.55 ± 0.08	0.49 ± 0.06

^1^ Diet-induced obese male 129S6 mice were fed western diet ad libitum or on a time-restricted feeding (TRF) protocol for 14 days. Liver tissues were harvested and frozen at sacrifice (day 15). Gene expression was determined by RT-qPCR. Relative mRNA expression was normalized to *Rplp0* and expression of each gene of interest was calculated using the standard curves method. Data are means ± SEM, * *p* < 0.05, ^†^ *p* = 0.21.

## Abbreviations

NAFLD	Non-alcoholic fatty liver disease
NASH	Non-alcoholic steatohepatitis
TRF	Time-restricted feeding
ER	Endoplasmic reticulum
ALT	Alanine transferase
AST	Aspartate transferase
SAA	Serum amyloid A
HE	Hematoxylin and eosin
FPLC	Fast performance liquid chromatography
FFA	Free fatty acids
VLDL	Very low density lipoprotein
LDL	Low density lipoprotein
HDL	High density lipoprotein
HOMA-IR	Homeostatic model assessment of insulin resistance
